# Harnessing Natural Killer Cells in Cancer Immunotherapy: A Review of Mechanisms and Novel Therapies

**DOI:** 10.3390/cancers13081988

**Published:** 2021-04-20

**Authors:** Frederique St-Pierre, Shailender Bhatia, Sunandana Chandra

**Affiliations:** 1 Division of Hematology Oncology, Feinberg School of Medicine, Northwestern University, Chicago, IL 60208, USA; frederique.st-pierre@northwestern.edu; 2 Division of Medical Oncology, Fred Hutchinson Cancer Research Center, University of Washington, Seattle, WA 98195, USA; sbhatia@uw.edu; 3 Division of Hematology Oncology, Robert H. Lurie Comprehensive Cancer, Northwestern University, Chicago, IL 60208, USA

**Keywords:** natural killer cells, targeted therapy, cancer immunotherapy, innate immunity

## Abstract

**Simple Summary:**

Cancer immunotherapy has mostly focused on harnessing adaptive immunity to promote the destruction of neoplastic cells by B and T lymphocytes. We are now increasingly recognizing the important role of the innate immune system in the body’s response to neoplastic cells, providing an opportunity for the development of innovative cancer treatments. Specifically, emerging therapies targeting natural killer (NK) cells, which are integral to the innate immune response, are being investigated for the treatment of various tumor types. Successful efforts would pave the way for entirely new immunotherapeutic strategies in cancer treatment, with numerous possible targets.

**Abstract:**

Natural killer (NK) cells are lymphocytes that are integral to the body’s innate immunity, resulting in a rapid immune response to stressed or infected cells in an antigen-independent manner. The innate immune system plays an important role in the recognition of tumor-derived stress-related factors and is critical to subsequent adaptive immune responses against tumor antigens. The aim of this review is to discuss mechanisms by which tumor cells evade NK cells and to outline strategies that harness NK cells for cancer immunotherapy. We discuss strategies to relieve the exhausted state of NK cells, recent therapies focused on targeting NK-cell-specific activating and inhibitory receptors, the use of cytokines IL-2 and IL-15 to stimulate autologous or allogeneic NK cells, and ongoing trials exploring the use of genetically modified NK cells and chimeric antigen-receptor-modified NK (CAR-NK) cells.

## 1. Introduction

Cancer immunotherapy has transformed the field of oncology, revolutionizing cancer care and leading to the development of new and improved standards of care [1]. As of yet, most successful immunotherapy agents, including monoclonal antibodies, immune checkpoint inhibitors, and chimeric antigen receptors (CARs), target the antigen-dependent adaptive immune system, with a large focus on targeting T-cells to enhance antitumor immunity [2]. The innate immune system, which acts in an antigen-independent manner to attack foreign or stressed cells, also harbors antitumor activity through various mechanisms and has the advantage of being inherently activated without requiring specific antigen presentation [3,4]. Natural killer (NK) cells are lymphocytes that are integral to the functioning of the innate immune system and play an important role in innate antitumor immunity [5]. Increasingly, NK cells are being recognized as a possible candidate for facilitating cancer immunotherapy.

In this review, we outline the role of NK cells in tumor suppression and expand on the mechanisms by which tumor cells can evade destruction by NK cells and the innate immune system. We outline novel therapeutic mechanisms for harnessing NK cell ant-tumor activity, including the role of NK-specific checkpoint inhibitors, enhancing NK cell activation through interaction with their activating and inhibitory receptors, the potential for adoptive NK cell transfer, including the novel CAR-NK agents, and advances in nanotechnology that could permit enhanced drug delivery and NK cytotoxicity to tumor tissues that are otherwise difficult to penetrate.

## 2. The Role of NK Cells in Innate Immunity and Tumor Suppression

Innate immunity is one of the two main immune strategies in vertebrates and the main immune strategy in invertebrates [5]. The innate immune system is the first line of defense against invading pathogens. It has the capacity to identify and destroy foreign (allogeneic) cells or stressed autologous cells (e.g., infected or neoplastic cells) in an antigen-independent manner. In other words, innate immune cells can recognize conserved features of pathogens that are not present normally in the host and are not specific to a particular pathogen. This makes this type of immune response more immediate, hence its role as the body’s first line of defense. Innate immunity also serves the important role of activating the second arm of the immune system, the adaptive immune system, through antigen presentation [3,4]. The innate immune response will either succeed in clearing foreign pathogens or containing them while the adaptive response develops [5]. 

The innate immune system is comprised of a variety of cells, including basophils, dendritic cells, eosinophils, Langerhans cells, mast cells, macrophages, monocytes, neutrophils, and NK cells [4]. The NK cell is a lymphocyte that is cluster-of-differentiation (CD)56-positive and CD3-negative (unlike T-lymphocytes, which are CD3-positive). Once the NK cell identifies a foreign cell, it induces apoptosis through the release of preformed cytotoxic granules (perforin and granzymes) to mediate cytotoxicity. This mechanism is similar to that of T-cells in adaptive immunity, although NK cells do not require activation for the destruction of foreign cells [5]. 

NK cells regulate their cytotoxic activity through two types of receptors: activating and inhibitory receptors. The balance of stimulation of inhibitory and activating receptors determines NK cell activity against a circulating target. Activating receptors include (1) Ly49, a C-type lectin receptor, which identifies the “self” marker of major histocompatibility complex (MHC) class I [6]; (2) natural cytotoxicity receptors (NCRs) (NKp30, NKp44, and NKp46); (3) transmembrane proteins that bind viral and bacterial ligands as well as tumor cell ligands such as proliferating cell nuclear antigen (PCNA) [7]; and (4) CD16 (FcγIIIA), which binds immunoglobulin (Ig) G for antibody-dependent cell-mediated cytotoxicity (ADCC) [8]. Inhibitory receptors include (1) killer-cell immunoglobulin-like receptors (KIRs) [9]; (2) Ig-like extracellular domain receptors that bind to MHC-I to inhibit the destruction of normal circulating autologous cells [4,5,10]; (3) CD94/NKG2, a C-type lectin receptor which identifies a number of ligands, including the nonclassical subtype of MHC-I, human leukocyte antigen E (HLA-E) [11,12]; (4) Immunoglobulin-like transcript/leukocyte immunoglobulin-like receptor (ILT/LIR), an Ig-like receptor; and (5) Ly49, which also has an inhibitory isoform and binds to MHC-I [4,5,10]. 

**Figure 1:**
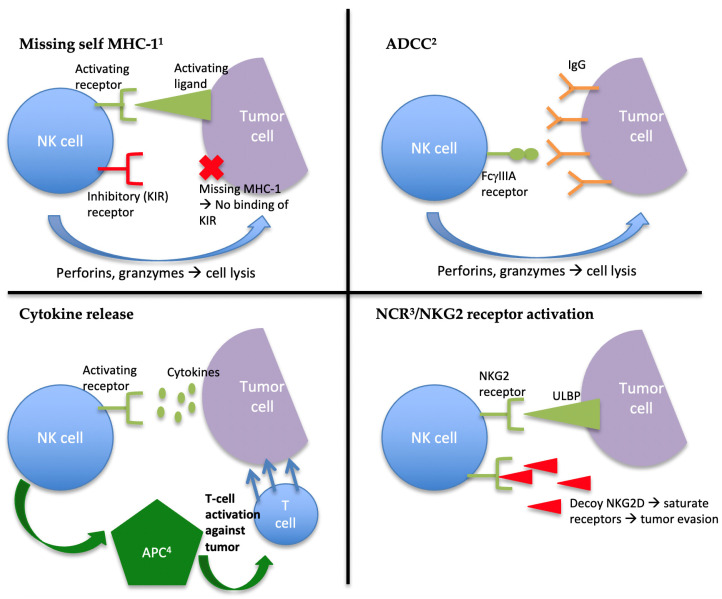
Major mechanisms of NK cell activation.^1^ Major histocompatibility complex-1; ^2^ antibody-dependent cell-mediated cytotoxicity; ^3^ natural cytotoxicity receptor; ^4^ antigen-presenting cell. (**A**): Absence of binding of MHC-1 to the KIR receptor on the NK cell leads to NK activation and tumor cell lysis. (**B**): The FcγIIIA receptor binds to the IgG receptor on tumor cells, resulting in NK cell activation. (**C**): Cytokines in the tumor microenvironment bind to activating receptors on the NK cell, leading to activation of APCs, which, in turn, target tumor cells for destruction by T-cells. (**D**): ULBP receptors on tumor cells bind to the NKG2 receptor on NK cells, leading to NK cell activation against the tumor cell. One mechanism for NK cell evasion by tumor cells is the production of decoy NKG2D ligands, which saturate the NKG2 receptors on the NK cell and prevent the binding of the ULBP receptor.

NK cells can identify foreign cells through multiple mechanisms (Figure 1). If the NK cell encounters a circulating cell that is missing the “self” MHC-I molecule, the inhibitory receptors that bind to MHC-I will fail to be activated, and the balance will shift towards the activation of the NK cell and the induction of apoptosis of the foreign cell [13]. This is an important mechanism, as cells that lose expression of MHC-I can evade the T-cell response from the adaptive immune system [14]. Another pathway for NK cell activation is through ADCC [8]. Infected cells are often opsonized with antibodies that subsequently bind to the CD16 receptor, resulting in NK cell activation. NK cells can also be activated by cytokine release, resulting in the release of interferon γ (IFNγ), tumor necrosis factor α (TNF-α), and several interleukins (IL) by the NK cell, which, in turn, engage antigen-presenting cells to activate the adaptive immune system [10]. 

With regards to antitumor activity, NK cells can be activated if tumor cells downregulate or express altered MHC-I molecules. They can also be activated through the binding of the NKG2 receptors to foreign ligands, typically expressed on tumor cells such as ULBP and MICA. Similarly, NCRs can bind to tumor cell ligands such as PCNA to induce apoptosis of neoplastic cells [10,15]. Nonetheless, tumor cells have developed mechanisms to evade destruction by NK cells; namely, certain tumor cells shed “decoy” NKG2D soluble ligands to saturate the NKG2D receptor on the NK cells to prevent the destruction of the tumor cells [16]. Moreover, tumor cells present a challenge for the NK cell response because they may express unaltered MHC-I molecules, in which case NK-cell-mediated destruction is inhibited by binding to KIR and NKG2 receptors. Furthermore, tumor cells may not be opsonized with antibodies for destruction through ADCC and may not result in significant inflammation and cytokine release to trigger NK cell activation [10,15]. 

Another limitation to the NK cell tumor response, which is also the case for T-cells, is their limited ability to reach tumor tissues [17]. Once in the tumor tissues, the tumor microenvironment itself suppresses NK cell activity. The presence of several immunosuppressive cytokines in the tumor microenvironment, such as transforming growth factor β (TGF-β), inhibits NK cell activity [18]. Other factors present in the tumor microenvironment have the potential to suppress NK cells, including prostaglandin E2 by inducing myeloid-derived suppressor cells (MDSCs) [19], adenosine through the binding of adenosine A2A receptors on NK cells [20], and indoleamine 2,3-dioxygenase (IDO) by catalyzing a reaction that inhibits the expression of NKp46 and NKG2 receptors [21,22]. Current NK-cell-targeted therapies aim to reactivate NK cells against tumor cells that have evaded innate immunity through the above mechanisms.

## 3. Current Advances in NK Cell Targeted Therapy

### 3.1. Checkpoint Inhibitors

NK cells have the intrinsic ability to target and destroy tumor cells that have lost or altered their expression of MHC-I molecules. However, tumor cells that manage to maintain normal expression of MHC-I can evade destruction by NK cells by binding to KIR receptors or NKG2 receptors, thereby inhibiting their activation [14,15,23]. The KIR and NKG2 receptors in this situation act as immune checkpoints and prevent the destruction of cancer cells. In order to “reactivate” NK cells against tumor cells that express normal MHC-I molecules, immune checkpoint inhibitors specific to these receptors have been developed and are currently under investigation [24,25]. 

A monoclonal antibody against the KIR receptor, lirilumab, has shown an adequate safety profile in a phase I study in patients with solid and hematologic malignancies [26]. Thirty-seven patients were included, and safety was established with no dose-limiting toxicity. Mild to moderate adverse events, including fatigue, infusion reaction, pruritus, and headache, were noted [26]. A phase I study also established the safety of combining lirilumab with ipilimumab, a cytotoxic T-lymphocyte-associated protein 4 (CTLA4) monoclonal antibody and immune checkpoint inhibitor [27]. Unfortunately, phase II trials to date have not demonstrated a definite improvement in outcomes with lirilumab. A phase I/II study combining lirilumab and nivolumab, a programmed cell death protein 1 (PD-1) checkpoint inhibitor, in patients with head and neck cancer, as well as phase II trials utilizing lirilumab as monotherapy or in combination with PD-1 and CTLA4 inhibitors, have not shown a significant improvement in outcomes with the use of KIR blockade [28,29,30,31]. Further phase II trials are still ongoing to determine the role of lirilumab in the treatment of other malignancies.

Monalizumab is a novel checkpoint inhibitor and monoclonal antibody targeting the NKG2A receptor, which is expressed by approximately 50% of circulating NK cells and 5% of CD8+ T-cells [32]. In vitro, monalizumab has shown antitumor activity in solid and hematologic malignancies [32,33]. A phase I study of monalizumab in patients with rheumatoid arthritis demonstrated minimal toxicity [34]. A phase I/II study involving 58 patients with advanced gynecologic malignancies also showed an adequate safety profile, and early results have shown short-term disease stabilization [35]. Interim results of a phase II trial combining monalizumab with the epidermal growth factor receptor (EGFR) inhibitor cetuximab in patients with squamous cell carcinoma of the head and neck have also shown a 31% objective response rate. Fatigue, pyrexia, and headache were the most common adverse events [32]. 

Importantly, although the PD-1 immune checkpoint was originally identified in T-lymphocytes, it has now been demonstrated that NK cells can also express the PD-1 receptor [36]. PD-1 checkpoint inhibitors likely enhance the innate immune system through the activation of NK cells in addition to the adaptive T-cell pathway. A phase II clinical trial is currently assessing the effect of pembrolizumab (anti-PD-1 antibody) in NK cell exhaustion in patients with advanced, unresectable stage III or IV melanoma through the measurement of NK cell exhaustion/proliferation by flow cytometry [37]. 

Finally, alternate checkpoint receptors expressed on T-cells, including T-cell immunoglobulin and mucin domain-containing protein 3 (TIM-3), lymphocyte-activation gene 3 (LAG-3), T-cell immunoreceptor with Ig and immunoreceptor tyrosine-based inhibitory motif domains (TIGIT), and CD96, can also be expressed on NK cells [37,38]. These receptors are upregulated in tumor-associated NK cells and lead to functional exhaustion of NK cells. Preclinical data show that blocking of these receptors with antibodies could be effective at reversing NK cell exhaustion and restoring antitumor immunity [37,38,39]. Phase I and II trials are ongoing to determine the safety and efficacy of inhibitors of these receptors in patients with solid and hematologic malignancies [15].

### 3.2. Targeting NK Cell Activation Through Cytokine Release

Several cytokines activate NK cells and promote their proliferation, including IL-2, IL-12, IL-15, IL-18, and IL-21 [22,40]. IL-2 has been explored as a potential therapy to promote NK cell activation, although regulatory T-cells express higher affinity to and are preferentially activated by IL-2, leading to the overall inhibition of NK cell proliferation and activity [41]. A mutant of IL-2 with increased affinity to NK cells, IL-2/15Rβ, has been constructed and is being evaluated in animal models [42]. IL-15 is another potential target, with the advantage of preferentially activating CD8+ T-cells and NK cells over regulatory T-cells [22]. A phase I clinical trial investigating the use of recombinant human IL15 (rhIL15) in patients with advanced solid tumors showed a profound expansion of circulating NK cells, and the drug was well tolerated [43]. Clinical trials are underway to further determine its efficacy in the treatment of cancer (in combination with obinutuzumab in relapsed/refractory chronic lymphocytic leukemia (CLL); in combination with ipilimumab and nivolumab in refractory cancers; in combination with alemtuzumab in adult T-cell leukemia; in combination with avelumab in relapsed/refractory mature T-cell malignancies; in combination with avelumab in renal cancer [22,44]. 

A superagonist of IL-15, ALT-803, has been developed with a longer half-life (25 h versus less than 40 min for rhIL15) and is hypothesized to result in enhanced ADCC in addition to prolonged cytokine stimulation of NK cells [22,45]. A phase I trial of ALT-803 combined with nivolumab in patients with nonsmall cell lung cancer showed good tolerability and objective responses in 6/21 patients [46]. Several ongoing clinical trials are evaluating its effectiveness in combination with monoclonal antibodies (nivolumab, rituximab) or adoptive transfer of NK cells, as discussed below [22].

IL-21 has also been shown to have an important effect on NK cell proliferation and survival in preclinical studies, and Phase I trials are underway to investigate its effect on NK cell expansion during the treatment of hematologic malignancies [47]. Recombinant IL-12 (rIL-12) demonstrated robust antitumor immunity in preclinical studies but was unfortunately associated with significant toxicities in early clinical trials. Alternate delivery methods for localized expression of IL-12 in the tumor microenvironment (TME) are showing some promise [48]. 

### 3.3. Promoting NK Cell ADCC

ADCC is an important mechanism by which NK cells target cells for destruction through the binding of CD16 (FcγIIIA) to IgG [8]. IgG monoclonal antibody therapy can be used to bind the CD16 receptor, which results in NK cell activation. Monoclonal antibodies that have been associated with promotion of ADCC include trastuzumab [49], pertuzumab [37], cetuximab [32], rituximab [50,51], and ofatumumab [52]. Additionally, an Fc-engineered CD133 antibody containing the S239D/I332E substitution has been evaluated in animal models for use in AML and is showing increased affinity to NK cells [53]. Similarly, transgenic chicken-derived anti-CD20 monoclonal antibodies have shown enhanced Fc effector function and greater anticancer potential compared to chimeric monoclonal antibodies against CD20, such as rituximab [54], in preclinical models. Finally, IL-2 and the high-affinity CD16 FcγIIIA (158V) allele were engineered into NK-92 cells (known as haNK), and the use of this engineered NK cell is under investigation in phase I and II trials, evaluating haNK alone, haNK in combination with anti-PD-L1 antibody avelumab, and haNK in combination with IL-15 [37,55]. 

Bispecific and trispecific antibodies, known as killer cell engagers (BiKEs and TriKEs), also participate in CD16-induced ADCC. These killer cell adapters are designed by fusing the Fv domain of tumor cell antigens with the Fv domain that binds to CD16. The goal is to promote the linkage, or “immunologic synapse”, of NK cells to tumor cells via the CD16 receptor [22,56,57]. BiKEs are comprised of two antibody fragments: one targeting the tumor antigen and the other targeting CD16 on NK cells. TriKEs are created by integrating IL-15 into the BiKE molecule, thereby increasing NK cell cytotoxicity [57]. These have shown promise in preclinical models, and a phase I/II clinical trial is currently being conducted to evaluate their safety and efficacy in the treatment of high-risk hematologic malignancies [57,58]. Recently, a nanoparticle-based trispecific NK cell engager (named nano-TriNKE) has been developed to target EGFR receptors on tumor cells and CD16 and 4-1BB on NK cells. This is in the preclinical phase but may become useful in the treatment of tumors that overexpress EGFR [59]. 

### 3.4. Adoptive NK Cell Immunotherapy

In order to restore and improve NK cell function in patients with malignancy, infusions of NK cells, either autologous or allogeneic (deemed “adoptive transfer”), have been studied. 

The first NK cell infusions were autologous peripheral blood cells that were extracted from the patient, stimulated with IL-2 in vitro, and infused back into the patient after lymphodepleting chemotherapy [60]. At higher doses, the IL-2-stimulated NK cells were found to have unacceptable toxicity, with severe capillary leak syndrome. At lower doses, toxicity was acceptable, although subsequent phase II trials failed to demonstrate efficacy [22,61].

Given the suboptimal efficacy of autologous NK cell infusions, the use of allogeneic NK cells was investigated. Clinical trials using T-cell-depleted hematopoietic cell transplantation from haploidentical donors in patients with AML showed an antileukemia response without significant graft-versus-host disease (GVHD) [62]. NK cytotoxicity was enhanced if there was a KIR-HLA class I mismatch, and the hypothesis for this is that NK cell “education” of self-HLA is a continuous process, and donor NK cells can acquire a new pattern of alloreactivity, allowing for enhanced antitumor response [61]. A trial involving patients with refractory AML-administered HLA-haploidentical NK cells, along with daily IL-2 following lymphodepleting chemotherapy, resulted in 26% of patients achieving a complete remission (CR) after NK cell adoptive transfer [61]. However, subsequent NK cell proliferation was halted by the interference with T-regulatory cells (T regs), promoted by their high affinity to IL-2. In subsequent trials, a recombinant IL-2 fusion protein with a lower affinity for T-regs improved NK cell expansion rate by 10% and resulted in 53% of patients achieving CR [61]. Furthermore, the use of NK cells ex vivo has been studied following hematopoietic stem cell transplant (HSCT) in high-risk myeloid malignancies. In a Phase I trial, expanded donor NK cells were infused after haploidentical HSCT with melphalan-based conditioning [63]. Of the 26 patients, 100% achieved engraftment, and the 3-year progression-free survival rate was 66%, with only one patient developing grade 3-4 GVHD [64].

Following these promising results, allogeneic NK cell transfer has been studied in multiple solid tumor types, including breast cancer, ovarian cancer, melanoma, renal carcinoma, colorectal cancer, hepatocellular cancer, and neuroblastoma [32,65,66]. Results have shown tolerability in early phase trials [67], although the challenge is in obtaining large numbers of functional NK cells, even with exposure to cytokines. To mediate this, artificial antigen-presenting cells have been developed to help expand NK cells ex vivo [22], and clonal NK lines, such as the NK-92 or haNK cell lines, have been developed to improve proliferation [55]. A Phase II study using off-the-shelf activated NK (aNK) cells in combination with ALT-803, an IL-15 superagonist, in Merkel cell carcinoma showed excellent tolerability of the combination and promising efficacy, with objective responses in 2 of 7 patients with refractory MCC, including reversal of PD-1 refractoriness in one patient [68]. This trial provides proof of concept for aNK-based therapy in MCC and supports an upcoming registrational trial (NCT03853317) using cryopreserved NK cells (not requiring on-site expansion) plus N-803 plus avelumab in patients with advanced MCC refractory to treatment with checkpoint inhibitors. In clinical trials, expanded and activated NK cell adoptive transfer is typically used in conjunction with lenalidomide, which has been shown to augment NK cytotoxicity, potentially by stimulating the expression of activating ligands on tumor cells [22]. A recent study is now suggesting the proteasome inhibitor bortezomib also augments NK cytotoxicity against multiple hematologic and solid malignancies in animal models [69].

In all, genetically modified NK cells represent a promising strategy for the optimization of off-the-shelf NK cell therapy. In addition to haNK cells, other targets are being explored for the development of genetically modified NK cells. In particular, TGF-β receptor 2 (TGFBR2) has been identified as an important mechanism for NK cell immune evasion by cancer cells in the TME. Overexpression of TGF-β in the tumor microenvironment is an important contributor to NK cell suppression in cancer. TGF-β is secreted by tumor cells, T-regs, and MDSCs. TGF-β downregulates activating receptors NKp30 and NKG2 on NK cells and reduces the expression of NKG2 ligands on tumor cells. Inhibition of the TGFBR2 receptor in combination with allogeneic NK cells has been shown to be effective at preventing resistance to NK cell antitumor activity in vitro [70,71]. A recent study has used CRISPR/Cas12a to generate induced pluripotent stem cells (iPSCs) with the knockout of the TGFBR2 gene to render NK cells resistant to TGF-β-mediated suppression. In vitro studies of this genetically modified iPSC are suggestive that this strategy could result in an “infinite supply” of NK cells with an enhanced tumor-killing function, making future off-the-shelf NK cell therapy more feasible [72]. Another target under investigation is the cytokine-inducible SH2-containing protein (CIS; encoded by the gene CISH), a negative regulator of IL2/IL15 signaling in NK cells. Knockout of CISH could, therefore, improve NK cell effector function [72,73]. Further in vivo studies will be necessary to confirm the role of these novel targets in clinical practice.

### 3.5. CAR-NK Therapy

One of the most important and promising avenues for NK-cell-directed therapy currently is the investigation of the potential for using CARs to potentiate NK antitumor activity. Their efficacy is being explored in different malignancies [74]. A CAR consists of an extracellular antigen-targeting domain, called the ectodomain, a transmembrane region, and one or more intracellular signaling domains. The ectodomain can be engineered to target a specific tumor-associated antigen [75]. This effectively results in genetic modification of immune cells [76]. CAR therapy has been mostly used to potentiate T-cell activity, although it is increasingly being studied in NK cells. The advantage of using CAR-NK over allogeneic NK cell transfer is the improved feasibility of obtaining a large number of functional NK cells, the capacity to target NK cells to a specific tumor antigen, and, potentially, an improved safety profile, with reduced risk for graft-versus-host disease. CAR-NK may also have the unique ability to achieve an antitumor response through both CAR-mediated direct cytotoxicity and the natural killing mechanisms of the NK cells in a CAR-independent manner [77].

The majority of current clinical trials with CAR-NK are using the NK92 cell line due to its improved proliferation ability in vitro, although peripheral blood mononuclear cells (PBMCs) and umbilical cord blood are also being used [77]. The target antigens commonly used in the CAR constructs include CD19, CD7, CD123, and CD33 to target primary leukemia cells and lymphoma cells [77,78], EGFR and human epidermal growth factor receptor 2 (HER2) to target glioblastoma cells and gynecologic cancers [79,80], MUCI to target solid tumors [41], ROBO1 for pancreatic cancer [41], CD5 to target malignant T-cells [81], FLT-3 to target leukemia cells harboring this mutation [82], and mesothelin to target ovarian cancer cells [83]; several phase I/II trials are currently underway [77]. Clinical results are mostly pending, although interim results from a phase I/II trial involving 11 patients with relapsed or refractory CD19-positive cancers, who underwent treatment with anti-CD19 CAR-NK, showed a 73% overall response rate, with 7 patients achieving complete remission. None of the patients developed major toxic effects [84]. Results from ongoing trials will help determine the future place CAR-NK will hold in cancer-directed therapy.

### 3.6. Enhancing Drug Delivery

Delivery of the activated NK cells to the tumor site continues to be a challenge, as penetration of immune cells into a solid tumor is often limited. The shelf life of NK cells after extraction from the patient or donor is also a concern. Nanoparticle technology is being explored for enhanced delivery of NK cells and other NK-activating drugs.

Extracellular vesicles are nano-sized vesicles composed of a lipid bilayer membrane, naturally secreted by several cell types, including NK cells. These vesicles will typically express specific proteins from their cell of origin (for example, CD56 in the case of NK cell vesicles) as well as lytic proteins such as perforin. These vesicles can act as the transportation medium for various proteins traveling from one cell to another and can serve to activate signaling pathways in target cells. These vesicles can be easily infused into the blood and can diffuse more readily across tissues, including tumor tissue. Specifically, they can traverse the blood–brain barrier and blood–tumor barrier. These vesicles are more easily stored than the immune cells themselves and can last up to 12 months “off-the-shelf”. These characteristics make extracellular vesicles an attractive option in the delivery of cancer therapies [22,57]. Extracellular vesicles isolated from activated NK cells and carrying their cytotoxic proteins have shown significant suppression of tumor growth in animal models [85,86]. These vesicles are also being investigated for use in drug delivery. A recent study has evaluated the use of these vesicles to codeliver a TGF-β inhibitor and selenocysteine to malignant breast tumors and demonstrated effective antitumor activity in mice [87]. Similarly, a nanoparticle that delivers small interfering RNA for the TGFBR2 receptor has been developed, resulting in the restoration of NK cell activation against malignant cells in the TME in vitro [88].

### 3.7. Overcoming Suppression by the Tumor Microenvironement

As discussed previously, TGF-β is an important contributor of NK cell suppression in the tumor microenvironment. Therapies that interrupt TGF-β signaling to restore NK cell activity against tumor cells are currently under investigation [37]. Fresolimumab is a TGF-β neutralizing monoclonal antibody that has shown good tolerability in a phase I trial with patients with advanced melanoma and renal cell carcinoma, as well as preliminary evidence of antitumor activity [89]. A clinical trial evaluating its use in combination with radiation therapy in metastatic breast cancer is underway (NCT01401062). Galunisertib is a TGF-β receptor type I (TGFBR1) inhibitor with the potential to restore NKG2 and NKp30 expression in activated NK cells [37]. A phase II study evaluating galunisertib in combination with sorafenib in patients with advanced hepatocellular carcinoma has shown an acceptable safety profile and a median overall survival of 18.8 months [90]. Therapies targeting downstream pathways of TGF-β, such as the SMAD2/3-related pathway, are also under investigation [37,76]. Table 1 summarizes the types of therapies interacting with NK cells discussed above.

## 4. Conclusions

NK cells are intricate to the innate immune system and allow for rapid recognition of cells that may otherwise go undetected by the adaptive immune system by recognizing and inducing apoptosis in cells with absent or altered MHC-I expression and through interaction with tumor cells through various activating and inhibitory receptors. Current FDA-approved immunotherapies target mainly T-cells and the adaptive immune system, although harnessing the innate immune system for cancer treatment, specifically exploiting the unique antitumor characteristics of NK cells, has a high potential for meaningful and groundbreaking therapeutic advances. Many clinical trials investigating various mechanisms for NK-cell-specific immunotherapy are currently in phase I or II trials. In addition to confirming the effectiveness of these novel therapeutic pathways, future studies will need to identify strategies to enhance the feasibility of NK-directed immunotherapy. Specifically, approaches to mediate the short shelf life of NK cells and incomplete penetration into the solid tumor microenvironment will need to be explored. In all, NK-cell-directed immunotherapy is a promising, novel therapeutic strategy that will deserve increasing attention in the years to come.

## Figures and Tables

**Table 1:** Summary of therapies interacting with NK cells.

Type of Therapy	Studied Agents	Mechanism/Target Receptor
Checkpoint inhibitors	Lirilumab Ipilimumab Nivolumab Pembrolizumab Monalizumab	mAb ^1^ targeting KIR mAb ^1^ targeting CTLA4 mAb ^1^ targeting PD-1 mAb ^1^ targeting PD-1 mAb ^1^ targeting NKG2A
Cytokine activation	IL-2 IL-2/15Rβ IL-15 ALT-803	IL-2 IL-2 (NK cells preferentially) IL-15 IL-15 (superagonist)
Enhancing ADCC^2^	Trastuzumab Pertuzumab Cetuximab Rituximab Ofatumumab	mAb associated with enhanced function of CD16 (FcγIIIA)
	NK-92 cell (haNK)	Engineered NK cell with IL-2 and the high-affinity CD16 FcγIIIA (158V) allele
	BiKEs ^3^ and TriKEs ^4^	Fuse Fv domain of tumor cell Ag ^5^ with Fv domain binding to NK cell CD16
Adoptive transfer	Autologous NK cell infusion Allogeneic NK cells (HLA-haploidentical) CAR-NK ^6^	NK cell repletion Targets specific tumor-associated Ag (CD19, CD7, CD5, CD123, EGFR, HER2, MUCI, ROBO1, mesothelin, etc.)
Nanoparticle technology	Nano-vesicles	Vesicles composed of lipid bilayer membrane with ability to carry NK-specific cytotoxic proteins and with potential for enhanced drug delivery
Optimizing the TME^7^	Fresolimumab Galunisertib	mAb neutralizing TGF-β TGF-βR1 inhibitor

^1^ Monoclonal antibody; ^2^ antibody-mediated cell-mediated cytotoxicity; ^3^ bispecific killer engagers; ^4^ trispecific killer engagers; ^5^ antigen; ^6^ chimeric antigen receptor; ^7^ tumor microenvironment.

## Data Availability

No new data were created or analyzed in this study. Data sharing is not applicable to this article.
